# Early intervention: determinants of neurodevelopmental outcomes in very preterm children

**DOI:** 10.1192/j.eurpsy.2025.1990

**Published:** 2025-08-26

**Authors:** A. Riera-Martín, A. Calvo, M. Estévez-Domingo, Á. Iruin-Sanz, M. Moreno-Íñiguez

**Affiliations:** 1 Department of Personality, Assessment and Clinical Psychology, Complutense University of Madrid, Madrid; 2 Basque Health System (Osakidetza), Biogipuzkoa Health Research Institute; 3 Basque Health System (Osakidetza), Donostia University Hospital; 4 Basque Health System (Osakidetza), Outpatient Mental Health Network of Gipuzkoa, San Sebastián, Spain

## Abstract

**Introduction:**

Based on previous studies, we already know that very preterm birth constitutes an important risk factor for neurodevelopmental disorders and psychopathology.

**Objectives:**

The present study aims to identify those variables that might impact the neurodevelopment of children born below 32 weeks gestation and 1.5 kg of weight, and their parents, in a three-year follow up.

**Methods:**

This is a prospective observational study implemented at Donostia University Hospital between January 2018 and December 2021. Inclusion criteria: newborns with gestational age < 32 weeks and/or birth weight < 1.5 kg, and their parents. Participants were 113 newborns, 87 mothers and 77 fathers. Children’s neurodevelopment was evaluated through the Bayley Scale of Infant and Toddler Development- Third Edition (Bayley-III) at their first, second and third years of life. Parents’ predictive variables, evaluated during the period of time in which their children were hospitalized in the Neonatal Intensive Care Unit (NICU), were parents’ mental health (postpartum depression, anxiety and general symptoms), postnatal bonding, and obstetric variables. To analyze the study’s dependent variables, step linear regression models (cognition, receptive and expressive language, fine and gross motor skills) at first, second and third year were employed.

**Results:**

Parents’ mental health, especially phobic anxiety in fathers and paranoid ideation in mothers, as well as postnatal maternal bonding, significantly predicted neurodevelopmental difficulties in children during their first three years of life. Children’s gestational age at birth and fathers’ obsessive-compulsive symptoms predicted neurodevelopmental difficulties in several models, although not as expected, in a counterintuitive manner. In terms of cognition and fine motor skills, the model’s predictors were not significant at age three.

**Conclusions:**

According to our results, we consider an early mental health intervention for parents of newborns in need of hospitalization at the NICU to be of major importance. This intervention should be implemented during the hospitalization period, focusing on both parents (father included) and addressing bonding issues from a preventive perspective.

**Disclosure of Interest:**

None Declared

